# Case Report: Transvertebral transposition of the spinal cord for recovery after paraplegia during kyphoscoliosis surgery

**DOI:** 10.3389/fneur.2022.915188

**Published:** 2022-12-08

**Authors:** Chao Chen, Zhen Zhao, Jing Li, Qiankun Xu, Weibin Zhong, Bingjin Wang, Lingwei Zhu, Cao Yang, Yong Gao

**Affiliations:** ^1^Department of Orthopaedics, Union Hospital, Tongji Medical College, Huazhong University of Science and Technology, Wuhan, China; ^2^Department of Orthopaedics, Hefeng Central Hospital, Enshi, Hubei, China; ^3^Department of Neurosurgery, Union Hospital, Tongji Medical College, Huazhong University of Science and Technology, Wuhan, China; ^4^Department of Integrated Traditional Chinese and Western Medicine, Tongji Hospital, Tongji Medical College, Huazhong University of Science and Technology, Wuhan, China

**Keywords:** neurology, case report, spinal cord transposition, intraoperative neurophysiological monitoring, kyphoscoliosis, neurological dysfunction, experimental therapeutics spinal cord transposition, motor-evoked potentials

## Abstract

**Introduction:**

Neurological impairment during spinal deformity surgery is the most serious possible complication. When confronting intraoperative neurophysiological monitoring alerts, various surgical management methods such as the release of implants and decompression of the spinal cord are always performed. Transvertebral transposition of the spinal cord is rarely performed, and its role in the management of acute paraplegia is seldom reported.

**Case description:**

The authors present two patients with kyphoscoliosis who experienced neurological deficits and abnormal neurological monitoring intraoperatively or post-operatively that were detected during correction surgery. Acute paraplegia was confirmed by a wake-up test. Subsequent spinal cord transposition was performed. Intraoperative neurophysiological monitoring motor-evoked potentials (MEPs) and somatosensory-evoked potentials (SEPs) were performed to detect the changes during the process. After transvertebral transposition of the spinal cord, the MEPs and SEPs were significantly improved in both patients during surgery. The spinal cord function was restored post-operatively and recovered to normal at the final follow-up in two patients.

**Conclusion:**

This case demonstrated that instead of decreasing the correction ratio of kyphoscoliosis, transvertebral transposition of the spinal cord under intraoperative neurophysiological monitoring may be an alternative therapeutic strategy for acute spinal cord dysfunction caused by deformity correction surgeries.

## Introduction

Neurological impairment during spinal deformity surgery is the most serious possible complication, usually due to traction, extrusion, or hypoxic-ischemia of the spinal cord. Since the application of intraoperative neurophysiological monitoring (IOM), the changes of motor-evoked potentials (MEPs) and somatosensory-evoked potentials (SEPs) during surgery could indicate the spinal cord ischemic or mechanical injury with high efficiency (1, 2). When confronting IOM alerts, surgical management methods such as the release of implants and decompression of the spinal cord are always performed. Occasionally, decompression may require a wide-range resection of bone structures, and the spinal cord transposition may occur spontaneously (3).

The relocation of an angulated spinal cord out of its bony canal into a new position (transposition of the spinal cord) is a technically feasible operation. It was first used by Hyndman (3) to treat congenital kyphosis of the cervicothoracic junction with neurological dysfunction. This procedure can relieve spinal cord compression and improve the preoperative neurological dysfunction associated with spinal deformity in selected patients (4–9). However, to our knowledge, no report has been published in the English-language literature focusing on the effectiveness of spinal cord transposition for the recovery of acute paraplegia intraoperatively or post-operatively.

Herein, we present the therapeutic outcomes of two patients who underwent transvertebral transposition of the spinal cord after acute paraplegia during kyphoscoliosis surgery.

## Surgical technique and case description

**Figure 1:**
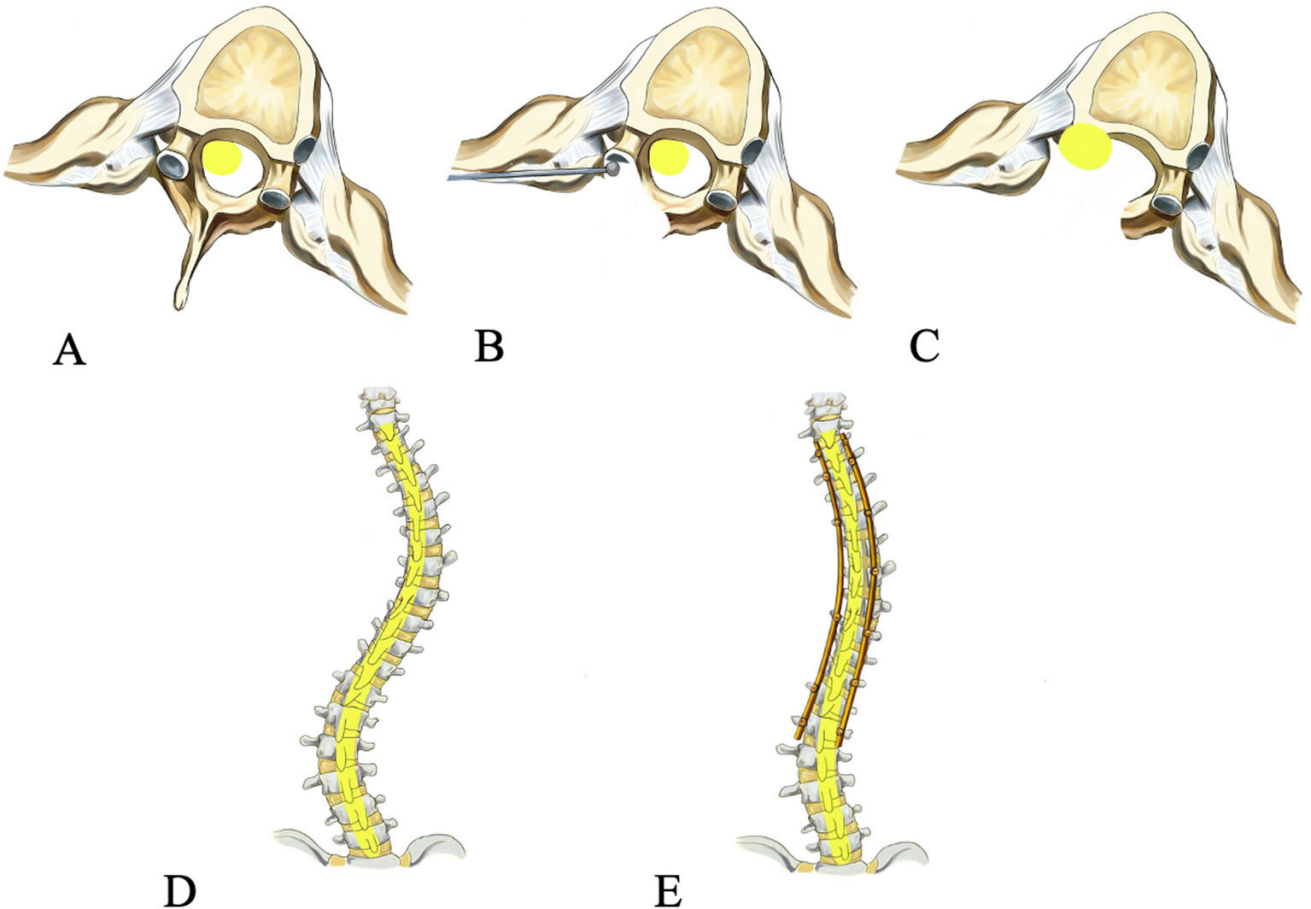
Operative procedure of spinal cord transposition. The spinal cord is compressed preoperatively **(A)**, the wall of the spinal canal and the pedicle on the concave side at the apex of the curve is drilled **(B)**, the spinal cord is transposed laterally and anteriorly **(C)**, posterior-anterior view preoperatively **(D)** and post-operatively **(E)**.

Two patients diagnosed with congenital kyphoscoliosis without preoperative neurological dysfunction were included. Written informed consent was obtained from both patients for publication of this case report and any accompanying details and images. During the correction surgery, spinal cord function was monitored using transcranial MEPs and SEPs. Abnormal SEPs were defined as a >50% decrease in amplitudes and/or a >10% increase in latencies than the baseline; abnormal MEPs were classified as a >50% decrease in amplitude than the baseline (10, 11). One patient experienced post-operative neurological deficits and abnormal neurological monitoring was detected during the revision surgery. The other patient suffered abnormal IOM. Transvertebral transposition of the spinal cord was performed in both patients to restore SEPs and MEPs signals successfully, and the operative steps are illustrated in Figure 1.

## Case 1

**Figure 2:**
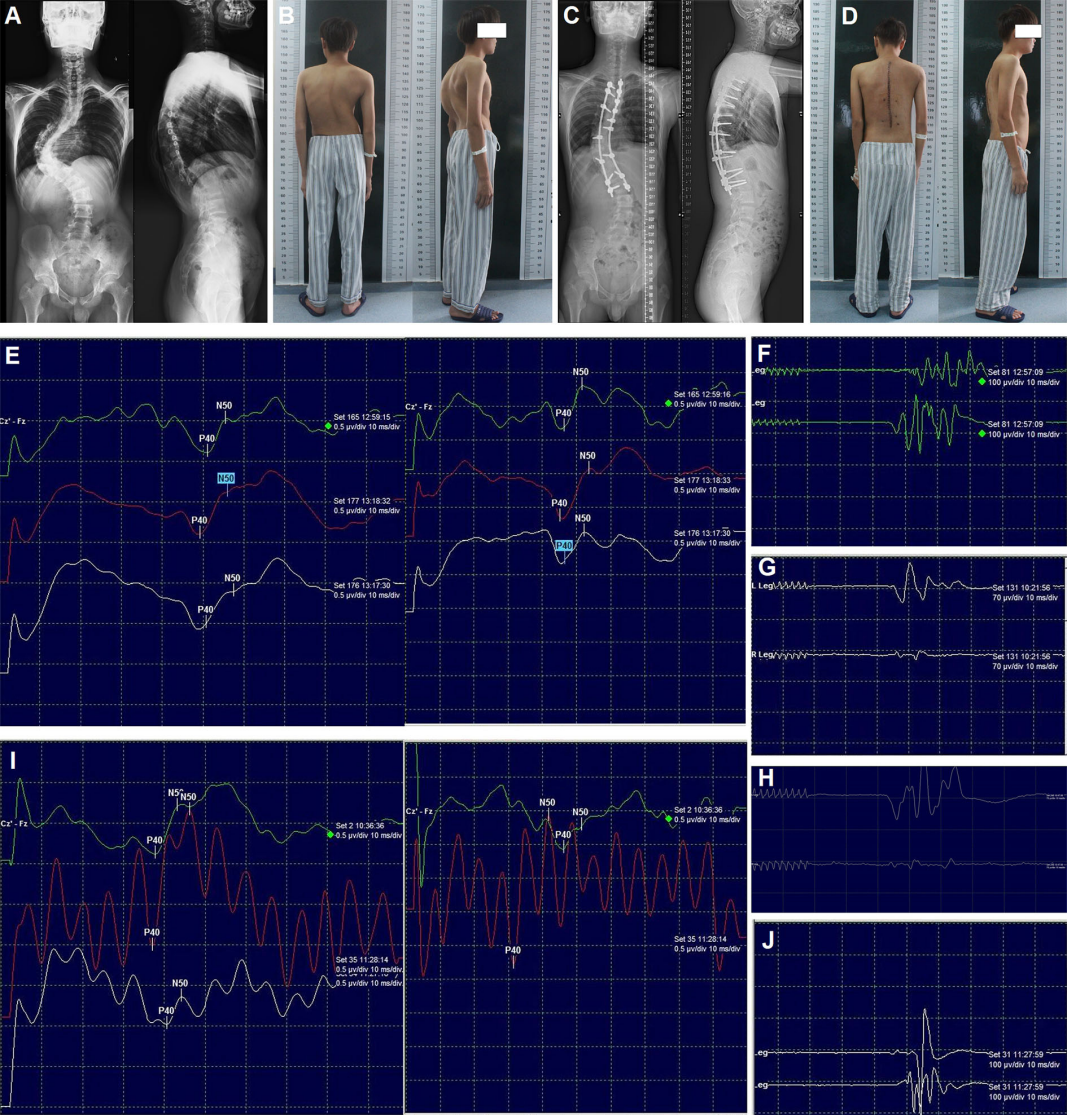
Radiography, appearance, and intraoperative neurophysiological monitoring of case 1. Preoperative radiography of the entire spine **(A)**, preoperative appearance in the posterior and lateral views **(B)**, post-operative radiography of the entire spine **(C)**, post-operative appearance in the posterior and lateral views **(D)**. SEPs **(E)** and MEPs **(F)** after correction; MEPs of the left **(G)** and right **(H)** lower limb after laminectomy; SEPs **(I)** and MEPs **(J)** after spinal cord transposition. MEPs: Motor-evoked potentials; SEP: Somatosensory-evoked potentials.

A 16-year-old boy presented with a 13-year history of congenital kyphoscoliosis. Brace treatment for 11 years was ineffective, and the curve progressed in recent years. Preoperative radiography revealed a Cobb angle of 105° and a flexibility index of 23% in the thoracic spine (Figure 2A). The preoperative appearance showed a rib hump, a thoracic trunk shift, and shoulder imbalance (Figure 2B). Preoperative computed tomography (CT) and magnetic resonance imaging (MRI) were performed, and no spinal stenosis or myelopathy or neurological dysfunction was found in the whole spine. Correction surgery, including multilevel Ponte osteotomies (12) and posterior spinal fusion of T3-L2, was arranged. During surgery, C-arm fluoroscopy showed a correction rate of 41% in the coronal plane and 47% in the sagittal plane.

No abnormal IOM signal was detected throughout the surgery (Figures 2E,F). However, 6 h post-operatively, the muscle strength was markedly decreased (right lower limb: grade 0; left lower limb: grade 3). The emergency CT scan did not reveal any misplacement of the pedicle screw, and post-operative intraspinal hematoma was suspected. One h later, the muscle strength was continually decreasing (right: grade 0; left: grade 0). Then, revision surgery was conducted immediately (7 h after primary correction surgery) with the rods and two screws at the apex removed and laminectomy performed, but no intraspinal hematoma was found. The wake-up test still demonstrated no voluntary movement in both lower limbs. Meanwhile (8 h after primary correction surgery), the SEPs decreased >70%, and the MEPs were totally lost. Simultaneously, the surgical team, including surgeons, neurophysiologists, and anesthesiologists, was taking steps to identify the possible causes according to a standard checklist for IOM alert (13). Finally, all the interference factors such as mechanical injury, pulling out of electrodes, low body temperature, muscle relaxant usage, low mean arterial pressure, and abnormal hemoglobin levels were excluded.

At last, we found that the spinal cord was stretched and closely attached to the wall of the spinal canal at the concave side with high tension, which could cause spinal cord compression. Therefore, we decided to remove the internal wall (including the lamina and the articular process, as well as the pedicles, heads of the ribs, and parts of the vertebral body) of the concave spinal canal of the three segments at the apex of the curve. Subsequently, the MEPs of the left and right lower limbs were recovered partially and slightly, respectively (Figures 2G,H). The entire wall of the concave spinal canal at the apex of the curve was then removed. When transvertebral transposition of the spinal cord was completed, and there was no compression or high tension of the spinal cord in the apical region, the bilateral SEPs and MEPs were progressively recovered (Figures 2I,J), and the correction surgery continued. Immediately after the patient woke up from the emergency revision surgery (12 h after primary correction surgery), his muscle strength recovered dramatically (right: grade 3; left: grade 4).

Additionally, the sensation of the lower limbs was almost normal, and the muscle strength was nearly normal before discharge. Post-operative radiography of the entire spine and appearance revealed satisfactory correction (Figures 2C,D). At the 3-month follow-up, he could return to normal life. No obvious loss of correction and neurological dysfunction was observed at the final follow-up (2 years).

## Case 2

**Figure 3:**
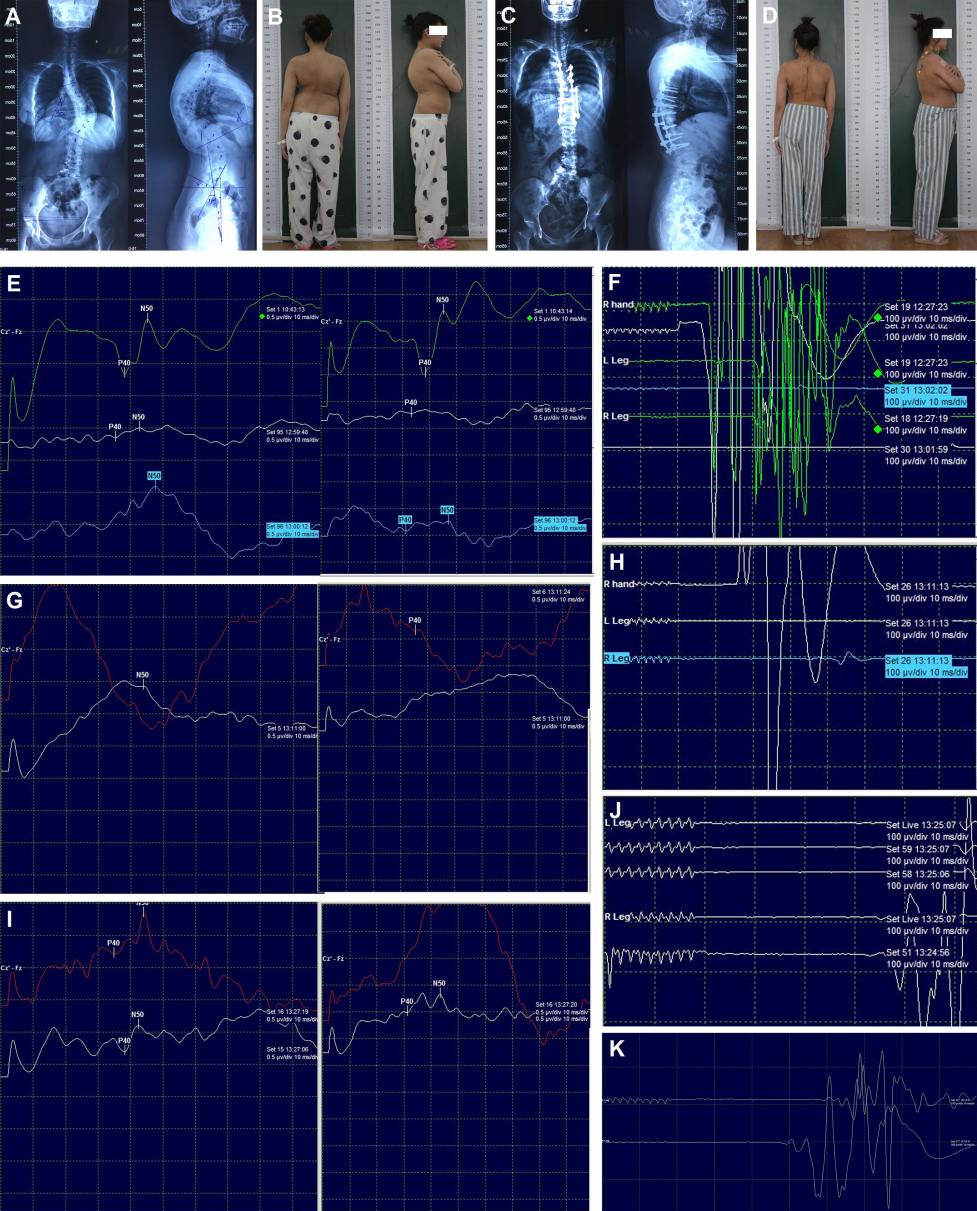
Radiography, appearance, and intraoperative neurophysiological monitoring of case 2. Preoperative radiography of the entire spine **(A)**, preoperative appearance in the posterior and lateral views **(B)**, post-operative radiography of the entire spine **(C)**, post-operative appearance in the posterior and lateral views **(D)**. SEPs **(E)** and MEPs **(F)** after correction; SEPs **(G)** and MEPs **(H)** after spinal cord transposition immediately; SEPs **(I)** and MEPs **(J)** 15 min later after spinal cord transposition; MEPs **(K)** at the end of surgery. MEPs: Motor-evoked potentials; SEPs: Somatosensory-evoked potentials.

A 19-year-old girl presented with a lifelong history of congenital kyphoscoliosis. Preoperative radiographic images of the entire spine exhibited a Cobb angle of 84° in the thoracic region with kyphosis of 55° (Figure 3A). The preoperative appearance showed the shoulder imbalance and trunk shift (Figure 3B). Preoperative CT and MRI were performed, and no spinal stenosis or myelopathy or neurological dysfunction was found in the whole spine. Correction surgery, including one-level pedicle subtraction osteotomy (14) and posterior spinal fusion, was scheduled.

Transpedicular screws were placed according to the preoperative plan. Asymmetrical pedicle subtraction osteotomy of T9 and posterior spinal fusion of T4-L1 were performed. During surgery, C-arm fluoroscopy showed a correction rate of 61% in the coronal plane and 42% in the sagittal plane. After the correction of the main thoracic curve, the amplitude of SEPs decreased significantly, and then the MEPs were lost (Figures 3E,F). The immediate wake-up test confirmed acute paraplegia. The surgical team then tried to identify the possible causes according to the checklist for IOM alert, and all the interference factors were excluded. Meanwhile, the removal of the rods and laminectomy was performed. Five min later, both the SEPs and MEPs were lost. Finally, high tension of the spinal cord was detected at the apex of the curve, and the spinal cord was closely attached to the wall of the concave spinal canal, which caused compression. The two pedicle screws at the apex on the concave side were then removed. Additionally, the wall of the concave spinal canal was removed and enlarged. Subsequently, the bilateral SEPs still showed no change, but the right MEPs were recovered about 15% (Figures 3G,H). About 15 min later (30 min after the IOM alert), the right MEPs were normalized, and the bilateral SEPs and left MEPs were partially recovered (Figures 3I,J). After transvertebral transposition of the spinal cord was completed, the correction surgery was started again. At the end of the surgery, the bilateral SEPs showed total recovery, and the left MEPs were almost recovered (Figure 3K).

The muscle strength and sensation of the right lower limb were almost normal, and the left lower limb had slight hypoesthesia and weakness (muscle strength: grade 4) before discharge. Post-operative radiography of the entire spine and appearance revealed satisfactory correction (Figures 3C,D). The muscle strength and sensation of both lower limbs had returned to normal at the 3-month follow-up. No obvious loss of correction and neurological dysfunction was observed at the final follow-up (18 months).

## Discussion

Transposition of the spinal cord was formerly used to treat patients with preoperative neurological dysfunction caused by congenital lateral kyphosis of the cervicothoracic junction (3), congenital cervicothoracic kyphoscoliosis (8), and kyphoscoliosis with spinal canal stenosis (9). Preoperative spinal cord compression could be released by the resection of bone structures that compress the spinal cord during the transposition of the spinal cord. Previously published cases (3–9) and our cases of spinal cord transposition are reviewed in Table 1. These previous studies indicated that spinal cord transposition was primarily aimed at eliminating preoperative spinal stenosis, which is different from our report. In addition, during the last two decades, IOM has been widely used to detect intraoperative spinal cord injury with high sensitivity and specificity. Transvertebral transposition of the spinal cord under IOM for the detection and treatment of acute paraplegia has not been reported previously.

**Table 1:** Literature review with summary of previously reported cases and our cases of transposition of the spinal cord.

**References**	**Age, sex**	**Diagnosis**	**Neurological function**	**Surgical approach**	**IOM**	**Prognosis**
Hyndman (3)	–	Kyphoscoliosis	Lower extremities paralysis and bowels or bladder dysfunction preoperatively	Transplantation of the spinal cord	No	Recovery
LOVE et al. (4)	28, male	Kyphoscoliosis secondary to Pott's disease	Numbness and weakness of both lower extremities preoperatively	Transplantation of the spinal cord	No	Recovery
	50, female	Pott's disease	Complete paraplegia preoperatively		No	Improvement
	11, male	Kyphoscoliosis	Weakness and hypesthesia of both lower extremities preoperatively		No	Improvement
	22, male	Kyphosis secondary to Pott's disease	Weakness of lower extremities preoperatively		No	Aggravation
	17, male	Scoliosis	Complete paraplegia preoperatively		No	Improvement
LOVE (5)	6 cases	Pott's disease	Paraplegia preoperatively		No	Recovery (3/6); revision surgery due to reactivation (1); little or no change (1); aggravation (1);
	65, male	Spinal injury	Paralysis from his waist down and bowels or bladder dysfunction preoperatively		No	No change
	53, male	Kyphoscoliosis	Paraplegia preoperatively		No	Improvement
	17, male	Kyphoscoliosis secondary to congenital hemivertebra	Paraplegia preoperatively		No	Recovery
	22, male	Kyphoscoliosis secondary to von Recklinghausen's neurofibromatosis	Paraplegia preoperatively		No	Marked improvement
	13, male	Kyphoscoliosis of the paralytic type	Paraplegia preoperatively		No	Marked improvement
Barber et al. (6)	16, male	Congenital scoliosis	Decreased sensory and motor function of both lower extremities preoperatively	Spinal cord transposition without fusion	No	Significant improvement
Feldman et al. (7)	17, male	Scoliosis	Decreased sensory and motor function of both lower extremities preoperatively	Spinal cord transposition with situ posterior fusion	No	Recovery
Shenouda et al. (8)	13, female	Kyphoscoliosis with two hemivertebrae	Right leg weakness, spinal pain and bladder dysfunction preoperatively	Spinal cord detethering and posterolateral decompression and transvertebral transposition of the spinal cord	No	Recovery
Novikov et al. (9)	15, female	Kyphoscoliosis	Paraparesis of the lower extremities preoperatively	Antero-lateral transposition of the spinal cord and posterior correction	SEP	Significant improvement
Current manuscript cases	16, male	Congenital kyphoscoliosis	Post-operative paraplegia	Multilevel Ponte Osteotomy, posterior fusion, and transposition of the spinal cord	SEP and MEP	Recovery
	19, female	Congenital kyphoscoliosis	Intraoperative paraplegia	Pedicle Subtraction Osteotomy, posterior fusion and transposition of the spinal cord	SEP and MEP	Recovery

IOM, intraoperative neurophysiological monitoring; SEP, somatosensory evoked potentials; MEP, motor evoked potentials.

For acute paraplegia intraoperatively or post-operatively, although various surgical techniques such as laminectomy, implant removal, decreasing correction rate, and even *in situ* fixation and extensive spine canal exploration were applied, they were rarely effective. Consistent with previous reports, the degree of post-operative functional recovery has often been disappointing regardless of treatment type (15, 16). In our two cases, we speculate that the acute neurological deficits may be related to severe and rigid kyphoscoliosis (Cobb angle >80° and flexibility < 30%) (17) and high tension of the spinal cord due to over-distraction during the correction. In case 1, the real-time IOM monitoring showed no abnormality during the operation, but delayed paralysis occurred 6 h after the operation; we considered whether there are problems concerning vascular insufficiency of the spinal cord due to over-distraction and ischemic edema of the spinal cord which may develop slowly and constantly. In case 2, the IOM alert developed immediately after the correction of the main thoracic curve, so we also wondered if there is undetected bony compression on the concave side or over-distraction to the spinal cord. In fact, during the surgery, all the aforementioned rescue surgical techniques were used but were ineffective in these two cases.

Since 1947, some studies have described that transposition of the spinal cord is performed to eliminate preoperative spinal stenosis caused by angulation deformity, release spinal cord compression, and improve preoperative neurological deficits (3, 6, 8, 9). The surgical technique of spinal cord transposition involved the removal of a part of the bone structure at the concave side, releasing the spinal cord compression, and preventing neurological deficits. In our study, transvertebral transposition of the spinal cord was eventually used to eliminate the spinal cord compression caused by kyphoscoliosis corrective surgery and to successfully recover the intraoperative or post-operative neurological deficits.

From these two cases, to prevent acute or delayed paralysis in such stiff curves, we must evaluate whether decompression is enough and if there is over-distraction or other problems concerning vascular insufficiency. Management strategy may include implant removal, laminectomy, decreasing correction rate, extensive spine canal exploration, and even transvertebral transposition of the spinal cord. If no compression to the spinal cord was found during the revision surgery, taking measurements to release the spinal cord tension may be an alternative method.

IOM, as an effective method to prevent neurological deficit, is used in most cases of spine surgery. IOM could dynamically monitor spinal cord function in real-time and indicate intraoperative neurological damage at an early stage, and catastrophic complications can be minimized if the adverse event is reversed promptly (18, 19). In our cases, the IOM alerts were checked by a standard checklist and confirmed by a wake-up test. After transvertebral transposition of the spinal cord, the IOM signals recovered, which objectively reflected the recovery of neurological dysfunction and confirmed the effectiveness of spinal cord transposition. However, our small sample size is a limitation. Hence, further studies are required in the future.

## Conclusion

In our study, transvertebral transposition of the spinal cord was eventually used to eliminate the spinal cord compression and to recover the intraoperative or post-operative neurological deficits successfully. This case demonstrated that instead of decreasing the correction ratio of kyphoscoliosis, transvertebral transposition of the spinal cord under IOM may be an alternative therapeutic strategy for acute spinal cord dysfunction caused by deformity correction surgeries.

## Data availability statement

The original contributions presented in the study are included in the article/supplementary material, further inquiries can be directed to the corresponding authors.

## Ethics statement

Ethical review and approval was not required for the study on human participants in accordance with the local legislation and institutional requirements. Written informed consent to participate in this study was provided by the participants' legal guardian/next of kin. Written informed consent was obtained from both patients for publication of this case report and any accompanying details and images.

## Author contributions

Conception and design and critically revising the article: CY, YG, and CC. Acquisition of data: LZ, ZZ, and QX. Analysis and interpretation of data: BW, WZ, and YG. Drafting the article: CC, JL, and ZZ. Approved the final version of the manuscript on behalf of all authors: YG. All authors have read and approved the manuscript in its current form.

## References

[1] Gonzalez AA Jeyanandarajan D Hansen C Zada G Hsieh PC. Intraoperative neurophysiological monitoring during spine surgery: a review. Neurosurg Focus. (2009) 27:E6. 10.3171/2009.8.FOCUS09150 19795955

[2] Deletis V Sala F. Intraoperative neurophysiological monitoring of the spinal cord during spinal cord and spine surgery: a review focus on the corticospinal tracts. Clin Neurophysiol. (2008) 119:248–64. 10.1016/j.clinph.2007.09.135 18053764

[3] Hyndman O. Transplantation of the spinal cord: the problem of kyphoscoliosis with cord signs. Surg Gynecol Obstet. (1947) 84:460–4. 20289163

[4] Love JG Erb HR. Transplantation of the spinal cord for paraplegia secondary to Pott's disease of the spinal column. Arch Surg. (1949) 59:409–21. 10.1001/archsurg.1949.01240040417004 18138055

[5] Love JG. Transplantation of the spinal cord for the relief of paraplegia. AMA Arch Surg. (1956) 73:757–63. 10.1001/archsurg.1956.01280050025006 13361733

[6] Barber JB Epps CH. Antero-lateral transposition of the spinal cord for paraparesis due to congenital scoliosis. J Natl Med Assoc. (1968) 60:169–72. 5661189 PMC2611496

[7] Feldman MD Bridwell KH Sheridan JJ. Hyndman-Schneider procedure for paraplegia caused by a sharp, angular scoliosis: a case report and a review of the literature. J Spinal Disord. (1993) 6:76–82. 10.1097/00002517-199302000-00015 8439722

[8] Shenouda EF Nelson IW. Nelson RJ. Anterior transvertebral transposition of the spinal cord for the relief of paraplegia associated with congenital cervicothoracic kyphoscoliosis technical note. J Neurosurg Spine. (2006) 5:374–9. 10.3171/spi.2006.5.4.374 17048777

[9] Novikov VV Vasyura AS Lebedeva MN Mikhaylovskiy MV Sadovoy MA. Surgical management of neurologically complicated kyphoscoliosis using transposition of the spinal cord: case report. Int J Surg Case Rep. (2016) 27:13–7. 10.1016/j.ijscr.2016.07.037 27521778 PMC4983149

[10] Park JH Hyun SJ. Intraoperative neurophysiological monitoring in spinal surgery. World J Clin Cases. (2015) 3:765–73. 10.12998/wjcc.v3.i9.765 26380823 PMC4568525

[11] Thirumala PD Huang J Thiagarajan K Cheng H Balzer J Crammond DJ. Diagnostic accuracy of combined multimodality somatosensory evoked potential and transcranial motor evoked potential intraoperative monitoring in patients with idiopathic scoliosis. Spine. (2016) 41:E1177–84. 10.1097/BRS.0000000000001678 27172278

[12] Geck MJ Macagno A Ponte A Shufflebarger HL. The Ponte procedure posterior only treatment of Scheuermann's kyphosis using segmental posterior shortening and pedicle screw instrumentation. J Spinal Disord Tech. (2007) 20:586–93. 10.1097/BSD.0b013e31803d3b16 18046172

[13] Ziewacz JE Berven SH Mummaneni VP Tu TH Akinbo OC Lyon R. The design, development, and implementation of a checklist for intraoperative neuromonitoring changes. Neurosurg Focus. (2012) 33:E11. 10.3171/2012.9.FOCUS12263 23116091

[14] Schwab F Blondel B Chay E Demakakos J Lenke L Tropiano P. The comprehensive anatomical spinal osteotomy classification. Neurosurgery. (2014) 74:112–20. 10.1227/NEU.0000000000000182o 24356197

[15] Hamilton DK Smith JS Sansur CA Glassman SD Ames CP Berven SH. Rates of new neurological deficit associated with spine surgery based on 108,419 procedures: a report of the scoliosis research society morbidity and mortality committee. Spine. (2011) 36:1218–28. 10.1097/BRS.0b013e3181ec5fd9 21217448

[16] Qiu Y Wang S Wang B Yu Y Zhu F Zhu Z. Incidence and risk factors of neurological deficits of surgical correction for scoliosis: analysis of 1373 cases at one Chinese institution. Spine. (2008) 33:519–26. 10.1097/BRS.0b013e3181657d93 18317197

[17] Zang L Hai Y Yuan S Su Q Yang J Guan L. Distal adding-on and risk factors in severe and rigid scoliosis. Spine. (2017) 42:160–8. 10.1097/BRS.0000000000001684 27172285

[18] Raynor BL Padberg AM Lenke LG. Failure of intraoperative monitoring to detect post-operative neurologic deficits: a 25-year experience in 12,375 spinal surgeries. Spine. (2016) 41:1387–93. 10.1097/BRS.0000000000001531 26913466

[19] Thirumala PD Bodily L Tint D Ward WT Deeney VF Crammond DJ. Somatosensory-evoked potential monitoring during instrumented scoliosis corrective procedures: validity revisited. Spine J. (2014) 14:1572–80. 10.1016/j.spinee.2013.09.035 24361128

